# Performance Evaluation of Knitted and Stitched Textile Strain Sensors

**DOI:** 10.3390/s20247236

**Published:** 2020-12-17

**Authors:** Kaspar M.B. Jansen

**Affiliations:** Emerging Materials Group, Department Industrial Design Engineering, Delft University of Technology, 2628 DE Delft, The Netherlands; k.m.b.jansen@tudelft.nl

**Keywords:** textile strain sensors, conductive yarns, knitted sensor, stitched sensor, performance evaluation

## Abstract

By embedding conductive yarns in, or onto, knitted textile fabrics, simple but robust stretch sensor garments can be manufactured. In that way resistance based sensors can be fully integrated in textiles without compromising wearing comfort, stretchiness, washability, and ease of use in daily life. The many studies on such textile strain sensors that have been published in recent years show that these sensors work in principle, but closer inspection reveals that many of them still have severe practical limitations like a too narrow working range, lack of sensitivity, and undesired time-dependent and hysteresis effects. For those that intend to use this technology it is difficult to determine which manufacturing parameters, shape, stitch type, and materials to apply to realize a functional sensor for a given application. This paper therefore aims to serve as a guideline for the fashion designers, electronic engineers, textile researchers, movement scientists, and human–computer interaction specialists planning to create stretch sensor garments. The paper is limited to textile based sensors that can be constructed using commercially available conductive yarns and existing knitting and embroidery equipment. Within this subtopic, relevant literature is discussed, and a detailed quantitative comparison is provided focusing on sensor characteristics like the gauge factor, working range, and hysteresis.

## 1. Introduction

Garments are intimate, close to the body and have a natural potential for collecting and monitoring body-related signals. Whereas in the early research of electronic textiles (or e-textiles), off-the-shelf sensors, electronic parts, and connecting wires were simply added to the textile, recent developments now allow for an almost complete integration of sensor and actuator functions in a textile [1,2]. Garments with well integrated electronics can be personalized, are comfortable, unobtrusive, and do not show visible connecting wires and sensor electrodes. Such functional clothing therefore has a much higher chance of being accepted to be worn in everyday life situations. In the near future stretch sensors will be embedded into everyday objects like pillows and car seats and we will use them during our fitness workouts, to monitor and correct our posture or during our virtual reality gaming. Apart from that, strain sensor garments are particularly beneficial for rehabilitation purposes where there is an urgent need for monitoring the recuperation of impaired body kinematics in situations of daily life activities [3]. Although textile strain sensors are now being evaluated in clinical rehabilitation settings, they still seem to lack sufficient resolution [4].

The era of smart textiles started with the introduction of conductive yarns and fabric at the end of last century which enabled it to integrate sensors and interconnections in garments in an unobtrusive way [2,5]. Conductive yarns are usually blending of traditional textile fibers and thin metal filaments like stainless steel, copper, or metal coated fibers consisting of a polymer core and a thin metal cladding (usually silver). Because of the blending with traditional textile fibers these conductive yarns still have their textile like properties and appearance. Current yarn manufacturing processes enable the production of yarns with non-conductive filaments wrapped around conductive core fibers. Such composite yarns have a fully textile appearance and are electrically insulated at the outside, and thus may prove useful as conductor lines in future e-textile applications [6,7]. An alternative way to achieve insulation is by coating the conductive filaments with micrometer thin polyurethane (enamel) layers. These types of insulated conductive yarns are however still not widely commercially available at present. 

Reviews on wearable and flexible sensors in general are given by [1,2,8,9,10,11,12,13], whereas [14,15,16] more particularly focus on textile strain sensors. Textile compatible strain sensors can be produced in three ways: (1) by integrating prefabricated stretchable sensor yarns in a garment; (2) by coating an existing fabric surface with a conductive substance, and (3) by integrating loop structures of conductive (non-stretchable) yarns in a textile fabric. In the latter case the resistance changes result from changes in contact resistance between loops of the conductive yarns during stretching. Sensor yarns or fibers can be manufactured by coating or dyeing yarns with a conductive layer or by embedding conductive particles in a stretchable polymer matrix [14,15,16]. In both cases the yarn resistance changes when stretched and sensor garments can be constructed by integrating them into the textile. Examples are the coating of stretchable yarns with carbon nanotubes [17], polypyrrole [18] or PEDOT [19], wrapping them with graphene film [20,21] or dyeing with silver nano crystal precursor solution [22]. Composite yarns were fabricated by adding e.g., carbon black [3], carbon nanotubes [23] or by blending with PEDOT solution [24]. Most of these sensor yarns show a high sensitivity, stretchability and good cyclic behavior. Although these yarns thus show high potential for future use as textile sensors, they are still in a relatively early research stage and require dedicated and well controlled processing, with the recently published kilometer scale conductive yarn sensor as the notable exception [24]. Coated fabrics (as studied in e.g., [25,26,27,28]) on the other hand have a lower sensitivity and tend to wear during use [14]. The advantage of the third category, the embedding of conductive loop structures in fabrics, is that the technology is directly accessible since it can be manufactured in mass production with standard equipment and uses commercially available types of yarns. 

The purpose of this review is therefore to present an overview of textile strain sensors that can be constructed using commercially available conductive yarns with standard textile manufacturing methods like knitting and stitching or embroidery. The paper is subdivided in 6 sections. In Section 2 and Section 3 the basics and terminology used in the fields of textile engineering and sensors are discussed. Next, in Section 4, the studies on knitted and stitched sensors are reviewed with a focus on the types of conductive yarns, stitches and substrate materials that were used. A detailed performance evaluation of the discussed sensors is treated in Section 5, while the main conclusions are summarized in Section 6.

## 2. Textile Basics and Terminology

Yarns can be combined to fabric structures by technologies like weaving and knitting. In weaving horizontal and vertical yarns are interlaced to form a fabric layer with a strong and deformation resistant structure. During knitting, on the other hand, consecutive rows of yarns are looped together to form a fabric structure which in principle consists of a single yarn. Knitted fabrics allow for a much higher comfort level because of its breathability and inherent ability to adapt conform to the human body shape without introducing pressure points. The fact that knitted garments stretch and are tight fitting makes them the ideal candidate as a platform to embed textile strain sensors.

### 2.1. Knitting Basics

**Figure 1:**
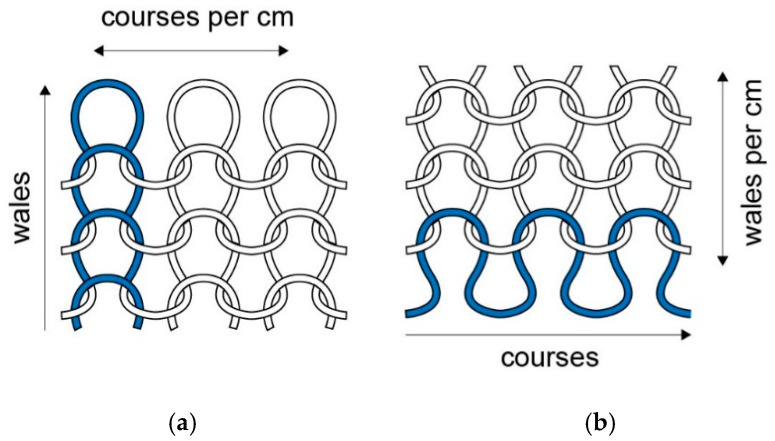
Single jersey knit stitch showing (**a**) course and (**b**) wale direction and their density (number per unit length).

The most common knitting technique is weft knitting in which horizontal rows of loops (called courses) are interconnected. With the consecutive addition of courses the knitted fabric grows in vertical direction. With the less common warp knitting method, however, loops are added to a vertical column of stitches which makes the fabric grow in horizontal direction. With modern flatbed knitting machines it is possible to integrate warp knitted vertical structures of (functionalized) yarns into a weft knitted base structure using special intarsia needles. A wale is a vertical column of loops produced by the same needle in a weft knitted structure, knitted at successive cycles (Figure 1a). Courses are rows of loops across the width of a fabric and are produced in the same knitting cycle (Figure 1b).

Computerized knitting machines have the possibility to make a wide variety of stitches. The single jersey stitch (or plain stitch) is the most standard one (Figure 1). A less dense fabric can be created by using only half of the needles. Other typical stitch types include the interlock stitch, Milan stitch and rib stitches. More details can be found in textbooks on knitting technology [29,30]. Each type of stitch has its own electromechanical behaviour and the selection of stitch type is therefore one of the important design parameters in the development of knitted strain sensors.

During the knitting process conductive yarns can be introduced as single yarns or co-knitted with a non-conductive support yarn in either the standard way or with the so-called plated knitting (vanisè) technology. In the former technique the conductive yarn is wrapped around the support yarn such that both the top and bottom face of the fabric are conductive, whereas with plated knitting the upper and lower yarns are kept separate, resulting in a fabric element with both a conductive and a non-conductive face. Such a separation of functional layers can also be obtained by double knitting. In that way pocket structures consisting of two distinct layers can be made [29]. An important consideration for the knitting with functional yarns is the matching of the yarn thickness with the machine gauge or the needle spacing of the knitting machine (usually given as the number of needles per inch). The higher the machine gauge, the thinner the yarns should be. The yarn thickness is expressed in terms of its linear density: the mass per unit length. Several systems and units are used. The commonly used tex, decitex (dtex) and denier (den) are defined as: tex = g/km, dtex = g/10 km and den = g/9 km. In textile engineering these linear yarn densities are often referred to as the yarn count numbers. Other definitions and conversion factors can be found in [29], p. 5. 

The dimensions (as well as the electrical characteristics) of knitted fabrics depend on how tight it is knitted. This is characterized by the stitch density, *S*, which is the total number of needle loops in a given area and is the product of the course count and the wale count (the number of courses and wales per unit length, see also Figure 1). The wale count is often expressed as wales per inch (*wpi*), the course count as courses per inch (*cpi*). A fabric with 12 wales per inch and 15 courses per inch is said to have a fabric count of 12 × 15. The stitch length is the length of yarn in a knitted loop. The longer the stitch length the more open and lighter the fabric. In weft knitted fabrics the course and wale densities are inversely proportional to this stitch length, *l*, [29]:

(1) $$cpi=\frac{k_{c}}{l},\;wpi=\frac{k_{w}}{l}$$

in which *k_c_* and *k_w_* are constants referring to courses and loops, respectively. With this the stitch density *S* becomes

(2) $$S=cpi*wpi=\frac{k_{c}k_{w}}{l^{2}},\;R=\frac{cpi}{wpi}=\frac{k_{c}}{k_{w}},$$

The ratio between the courses and wales per unit length is called the loop shape factor, *R*. According to Spencer the *k_c_* value varies between 5.0 and 5.5 whereas the loop shape factor tends to be always close to 1.3.

The planar size of knitted fabrics depends on the knitting compactness. The tightness Factor, *TF*, is defined as the area covered by the yarn in one loop relative to the total area occupied by that loop [30], p. 219:

(3) $$TF=S*l*d\sim \frac{\sqrt{tex}}{l}$$

For the scaling use is made of Equation (2) and the relation $\frac{m}{L}=10^{6}tex=\frac{\pi }{4}\rho d^{2}$. Note that in the Spencer book the proposed scaling is given as √(*tex*/*l*) which is inconsistent with the derivation above. In most plain fabrics the tightness factor ranges between 1.4 and 1.5 but note that for conductive yarns containing metal filaments with higher density this approximation may no longer hold. It is to be expected that this tightness factor will also affect the electrical contact properties. The tightness is of course also directly related to the porosity and breathability of a fabric. It can be varied by changing the NP setting of the knitting machine which determines the loop size. In practice, just knitted fabrics always have tension between the yarns which relaxes during movement and when wet. Therefore, the tightness of a fabric is always measured in the fully relaxed state, i.e., after 24 h washing in water at 40 °C followed by 1 h drying at 70 °C [29], p. 281. 

Other knitting parameters to consider are tension, take-down speed and cam speed [30], p. 217. According to [31] tension is the most important factor for knitting conductive yarn. It is the amount of distance that each needle pulls down after a knitting movement and controls the tightness of the stitches. Higher tension means looser stitches. 

**Figure 2:**
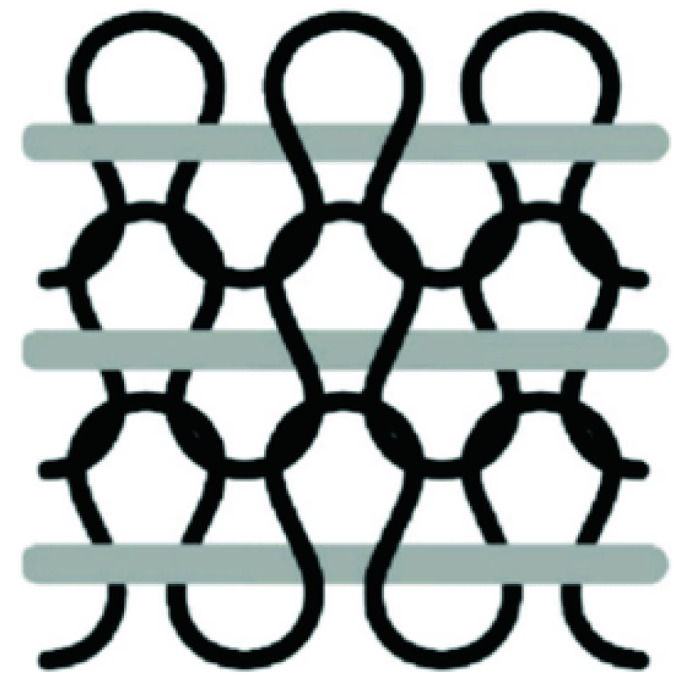
Inlaid yarns (gray) embedded in a double jersey structure [32].

With inlay knitting (Figure 2) an external (functional) yarn can be embedded in the knit structure which allows conductive external interconnect wires to be integrated in the garment as well as the construction of strain gauges consisting of a single meandering strain sensitive wire. In order to prevent discomfort and wrinkles during stretching, it is important to ensure that the stiffness of the inlay yarns is comparable to that of the base fabric.

### 2.2. Stitching and Embroidery

In a knitted fabric the functional yarn is part of the structure. However, with embroidery and stitching the (functional) yarns are attached to the surface of an existing fabric. Embroidered conductive yarn structures can also act as strain sensors and will therefore also included in this review. They offer a simple way to add sensors to an existing garment. 

**Figure 3:**
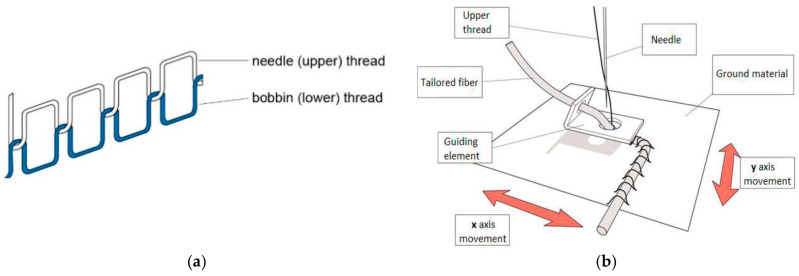
(**a**) Double lockstitch embroidery. The upper thread can be conductive; (**b**) Tailored fiber placement (TFP) embroidery; useful for fixating of functional fibers [34].

Figure 3a shows the structure of the basic double lockstitch in which and upper thread is fixated in the fabric by a lower thread supplied by the bobbin spool. Since both threads are confined to a single face of the fabric, the use of threads with different properties allows it to design patterns which are conductive at one face and insulate at the other face. A more complete overview of embroidery stitches can be found in [33]. Here we only mention the tailored fiber placement (TFP) stitch (Figure 3b) with which it is possible to fixate an external element like a hollow tube, optical fiber, or conductive filament to a fabric surface, similar to the yarn inlay technique used in knitting.

## 3. Strain Sensors

### 3.1. Sensor Types

Sensors are materials of which we can use the change of one of their properties (like electrical resistance) to detect and monitor the evolution of an external action (a force, deformation or, e.g., change in humidity). The strain sensors commonly used in mechanical engineering, for example, consist of a metal film pattern on a thin polymer substrate. When the substrate material deforms, the strain is transferred to the metal grid and the resistance change in the metal film is used to sense the deformation. Metal film strain gauges are therefore an example of strain-resistive type of sensors (i.e., a resistance change due to a deformation input). In literature also the term piezo-resistive is used for these type of sensors. In this work, however, we reserve the word piezo for the out-of-plane (squeezing) type of deformation as detected by pressure sensors and use the term strain-resistive to refer to in-plane deformations. The difference is important because sensors preferably should be selective: the strain-responsive sensors that we wish to design should be sensitive to in-plane deformation changes and as inert to pressure changes as possible.

The strain-resistive sensors discussed so far usually operate under constant current condition. As an alternative, it is also possible to observe the changes in the electrical impedance if an alternating current (AC) is applied. Impedance measurements can be done using the same sensor layouts as the common DC sensors [35] but also allows for new layouts. The distance between parallel conducting lines can for example be easily measured if one conductor line carries a carrier AC current and the induced current is picked up by the second conductor line [36]. Based on this a network of cm spaced carrier and sensor lines could be constructed, allowing for higher resolution strain mapping than is currently possible with DC based sensors. An additional advantage is that with impedance sensing data over a range of frequencies is obtained which can be used to filter out spurious behaviour.

### 3.2. Sensor Response Characterization

**Figure 4:**
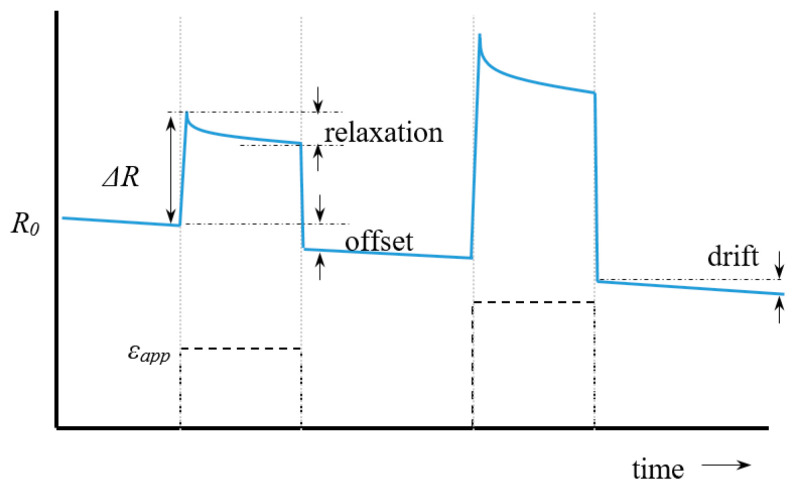
Schematic response of sensor after applied strain steps (black pulses).

Ideally a sensor signal should be stable over time, reproducible and linear with the input signal (a 10% input increase will then result in a 10% increase of the output signal). Metal film gauges are good examples of reliable, robust, and stable sensors which can be considered as a mature technology now. The textile based sensors that we wish to explore here are not. These sensors show hysteresis, are seldom linear, suffer from baseline drift and degrade over time. Moreover, details about this non-ideal behaviour are seldom reported in literature which complicates direct comparison. Before discussing reported sensor characteristics, we first outline the types of non-ideal sensor behaviour which may appear in experiments. A typical signal plot of a non-ideal sensor is shown in Figure 4. This figure shows how the resistance changes over time after application of two distinct strain steps, ε_app_ (shown as the dashed lines). 

After application of the strain the sensor response Δ*R* does not remain constant but slowly decreases with time (relaxation). In textile based sensors this is usually associated with the readjustment and sliding of yarn segments. The offset is the difference in signal before and after the pulse. The baseline is the measured response when the sensor is not loaded. It often slowly changes over time, which is denoted as drift. This drift can be either due to artefacts in the electronics or to structural relaxation of the yarn if the garment is under tension. Baseline changes may also occur during physical activity or changes in the environment (temperature, sweat, or humidity). Dynamic baseline drift describes how much the sensor signal at zero extension drifts over consecutive cycles; static baseline drift shows the change in sensor resistance during static conditions.

**Figure 5:**
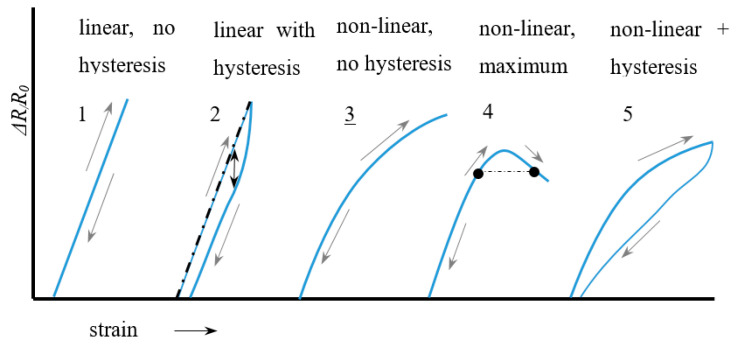
Non-linearity and hysteresis examples.

A direct consequence of the relaxation is that the signal during stretching differs from that during the subsequent retraction. This is called hysteresis and is shown in Figure 5 below. Non-linearity in itself is not a real problem regarding the signal interpretation since each measured Δ*R*/*R*_0_ value then still corresponds to a single strain value (case 3 in Figure 5). The only difference is then that the gauge factor (linearity constant) has to be replaced by a calibration curve or fit function. When, however, a maximum occurs (case 4) a measured Δ*R*/*R*_0_ value can correspond to two strain values. The practical range of the sensor (the working range) is then limited to the strain value at which the maximum occurs. In knitted and embroidered structures this type of response curve is often observed and is related to the stitch structure and the knit density. 

The sensitivity of a sensor is expressed as the gauge factor *GF*:

(4) $$GF=\frac{\Delta R/R_{0}}{\varepsilon }$$

The higher the response to a certain strain, the higher the gage factor (or gauge factor). A gauge factor of 1 means that every 10% elongation results in a 10% resistance increase. Note that the gauge factor is usually not defined as the initial slope but as the average value over a certain range. The transverse sensitivity, *TS*, is a number which indicates how sensitive the sensor is to transverse deformation with respect to length changes. It is defined as the ratio between the corresponding gauge factors:

(5) $$TS=\frac{GF\left(\mathrm{transverse}\right)}{GF\left(\mathrm{axial}\right)}$$

**Figure 6:**
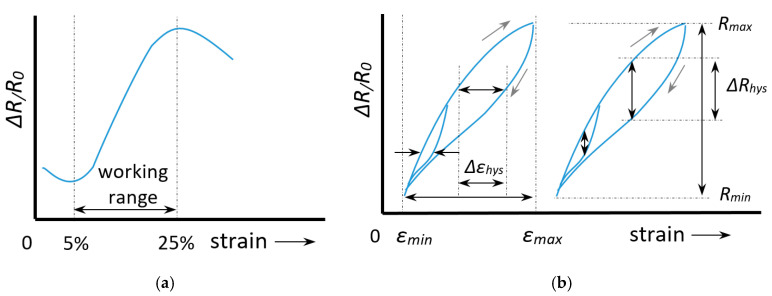
Definitions of: (**a**) Working range; (**b**) Horizontal and vertical hysteresis.

Ideally the transverse sensitivity should be close to zero. The working range can be defined as the strain range with a monotonic increase or decrease in the resistance-strain curve which unambiguously relates a strain value to a given resistance change. Figure 6 shows an example with a working range of 5–25%.

Hysteresis is the difference in signal measured during a forward and a backward movement and can be defined based on the relative deviation on the horizontal (strain) axis, or that on the vertical (resistance) axis (Figure 6b and Equation (6))

(6) $$H_{\varepsilon }=\frac{\Delta \varepsilon_{hys}}{\varepsilon_{max}-\varepsilon_{min}},\;H_{R}=\frac{\Delta R_{hys}}{R_{max}-R_{min}}$$

In the few studies that report the hysteresis error sometimes the deviation in resistance (vertical hysteresis) is chosen [37], whereas other times the horizontal hysteresis is used [38]. In practice, however, we are interested in how accurate we can obtain strain values from our measurements so it would make more sense to use the strain based definition, *H_ε_* to quantify hysteresis errors. Further, note that the amount of hysteresis strongly depends on the starting point. A sample which is deformed up to, say 40% will have much more yarn slippage and thus show a much larger hysteresis than a sample that is only 5% strained (see Figure 6). Part of this effect is already taken into account by scaling the hysteresis to the range maximum (Equation (6)), however, since yarn slippage (or relaxation) may be disproportionate at larger strains, it is a good habit to also mention the maximum strain when reporting hysteresis errors.

There are several types of experiments which are used to characterize a strain sensor. First of all, we have the step-up step-down or block pulse strain experiments as shown in Figure 4. Typically in these experiments the applied strain is increased stepwise in groups of three (e.g., three times 10% strain, followed by three 20% pulses, etc.). An alternative way is to increase the strain in an incremental, stepwise way (staircase profile). In both methods the gauge factor is evaluated by plotting the relative resistance change after a fixed time interval versus the applied strain. The results depend on the (arbitrary) choice of the time interval and the choice to take *R*_0_ as the resistance at the start of the first measurement or as the value just before the next strain step. In the third type of experiment the strain is applied at a constant speed and the resistance change versus strain curve is obtained as a direct result. If the upward curve is followed by a strain decrease step a hysteresis curve as shown in Figure 5 (case 2 and 5) is obtained. What is often not realized is that during such constant speed experiments also relaxation occurs and that there is more relaxation in a slow experiment than in a fast one. All results are thus, in principle, speed dependent, although [39] showed that for Electrolycra based sensors the velocity dependency was low. If a newly fabricated sensor is loaded to a certain strain for the first time, its behaviour often differs from subsequent loading cycles. Therefore, the first of the three block pulses in experiment type 1 described above is often discarded. The explanation is that during the first stretch microcracks occur (in metallic coated fibers) and that the fabric structure changes irreversibly. Experimentalists therefore often precondition their sensor by stretching it to the highest intended strain level before doing the actual experiment.

In some studies hysteresis and relaxation are compensated with dedicated data processing algorithms [40] or by comparison with extra sensors [41]. This is possible but goes at the expense of simplicity. A probable better way would be to redesign the sensors such that hysteresis is minimized and the maximum is shifted towards the end of the required sensor range (for skin deformation this is typically near 40% [3]).

### 3.3. Sensor Resistance

The resistance of an object increases with increasing length and decreases with increasing width and thickness. Equation (7) shows the formula used in literature for a rectangular body, a wire and a plane

(7) $$R=\rho_{V}\frac{L}{h\cdot W}=\rho_{L}L=\rho_{A}\frac{L}{W}$$

in which *ρ_V_* is the resistivity in Ω/m, *ρ_L_* = *ρ_V_*/*h* (Ω/m^2^) and *ρ_A_* = *ρ_V_*/A (Ω/sq) denote the length and area resistivity, respectively. The *ρ_L_* is the property used to characterize conductive wires and yarns, whereas *ρ_A_* is the resistivity property of a conductive fabric. Its units are in ohm but are usually expressed as Ω/sq (ohm per square) to differentiate it from the resistance itself (also in Ω). The ratio *L*/*W* is usually referred to as the aspect ratio.

## 4. Knitted and Stitched Strain Sensors

In this section we will discuss the manufacturing details (types of yarn and knitting parameters) and electromechanical behaviour of knitted and stitched structures, which are able to detect and monitor deformation. The focus will be on sensors which can be knitted with a computer controlled knitting machine and commercially available conductive yarns. Knitted fabric sensors can easily be stretched to over 100%, and such behaviour is indeed often reported. However, the applications that we have in mind are for on the body worn garments where strains are not larger than 35–45% [3]. For respiration monitoring the sensing range of interest is even smaller (up to 5%). While discussing the sensitivity and other properties we will therefore in the following limit ourselves to the first 40%. The ideal sensor properties for these type of applications can be formulated as [42]:

- Wide working range (up to 45% for measuring stretch on the human body)
- High enough gauge factor (sensitivity)
- Low hysteresis
- No signal drift
- Good repeatability (i.e., after multiple cycles and after washing)

### 4.1. Knitted Sensors

#### 4.1.1. Strain Sensitive Knitted Structures (1999–2009)

The number of published studies on knitted strain sensors in which details are given about the stretch strain behaviour and electrical responses are limited. One of the first studies showing the usefulness of textile based sensors is that of Farringdon [43]. They knitted 10 mm wide sensor strips from conductive fibers with a nominal resistance 1 MΩ/m. These sensors were sewn on a jacket to monitor limb and upper body motion and had a quite high gage factor (close to 17 for the first 20% elongation). Unfortunately no details about the conductive yarn and knitting parameters were given. Later on Vogl et al. [44] used the same idea to manufacture simple textile user interfaces by stitching conductive yarns on elastic fabric strip.

Another early study was that by Bickerton [45] who used conductive carbon fibers to construct a knitted fabric sensor with an initial resistance of about 90 kΩ which increased up to 460 kΩ at 30% stretch (*GF* = 14) and had a usable working range up to about 50%. He interpreted the shape of the resistance curve in terms of fiber slipping during extension and the associated increase in conducting path between contact points.

**Figure 7:**
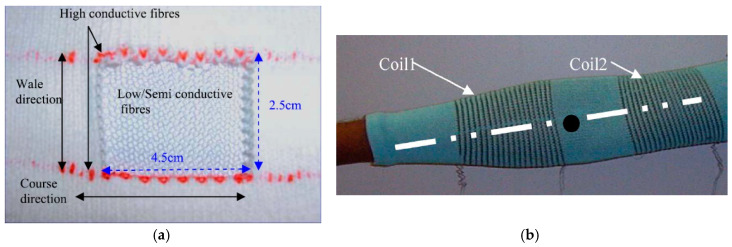
(**a**) Knitted sensor [35]; (**b**) Knitted coil sensors in arm sleeve [46].

The Advanced Textiles group of Tilak Dias (Nottingham Trent University) did much of the preliminary work on knitted textile sensors and claimed a first patent in this area [28]. Some details are given in [35] who describe relatively wide knitted sensor mesh structures of 45 mm (course) by 25 mm (wale) directions (Figure 7a). Their measurements show that these sensors mesh are much more sensitive in course direction (*GF* = 2.4 over the first 10% stretch) compared to wale direction (*GF* = 0.42, over the first 10%). The sensitivity quickly levels of after 10% stretch. No details about the yarn types are given however. In his 2006 study, Wijesiriwardana [46] also considered a different way of strain sensing by measuring the change in inductance between two knitted conductive coils after a linear or angular displacement. This technique could not only be used for respiration monitoring but also for angular movement monitoring on fingers and arms (Figure 7b). They claimed that these types of sensors are less sensitive to temperature drifts as well as to aging due to washes as compared to resistive transducers. These types of sensors can in principle be used both as linear and as angular sensor.

The possibility to continuously monitor the body kinematics of patients during daily life would be a breakthrough in the rehabilitation field [47]. The Pisa group in a collaboration with Smartex have been working on this since the early 2000s. Their early work considered sensing fabrics constructed by impregnating fabrics with conductive polymer or elastomer coatings [48,49]. Because of relatively low sensor accuracy, long response time, and hysteresis effects, the group then developed a series of knitted sensors based on a carbon filled nylon fiber (Belltron, [47,50] and references therein). The sensor discussed in [47] had a gauge factor of about 6.7, a working range of at least 10% and an acceptable hysteresis. In subsequent work they build and investigated several health care systems, as summarized in [51,52]. Sensors were fabricated by applying a conductive carbon filled silicone rubber coating to the fabric or by constructing knitted strain sensors. In [51] they presented a knitted sensor with gauge factor 0.75, high linearity (up to 18% strain) and low hysteresis. Their findings suggest that hysteresis is suppressed by applying the conductive yarn in an ordered structure. Although they were not clear how this is done, the most likely interpretation is that they achieved this by using the plated knitting technique (see Section 2.1).

**Figure 8:**
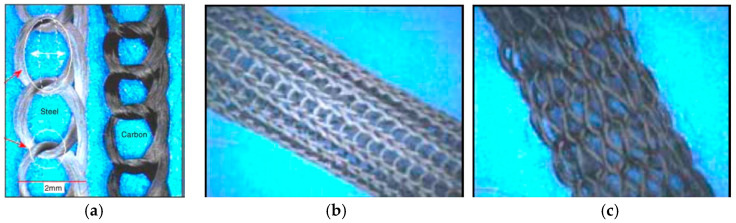
(**a**) Knitted single warp steel and carbon structures; (**b**,**c**) tubular structures [53].

Zhang [53] knitted heat resistant structures consisting of pure steel and carbon yarn bundles (Figure 8). They argued that the change in contact resistance between conductive wire loops is the key factor for the electromechanical response of knitted strain sensors. For the experiments, they used conductive yarns consisting of 120 stainless steel fibers of 30 μm diameter, knitted into a plain knitted fabric with wale and course densities of 26 and 40 units per 50 mm. Their results indicated a gauge factor of about −10 over the first 10% stretch. After 20% stretch the resistance almost dropped to zero, most probably since they used only pure stainless steel fiber yarns without support yarns. The main focus of their 2005 work [54] was to model the electromechanical behaviour of the knitted conductive networks shown in Figure 8. Their experimental data showed that such networks have a linear response up to 10% and a gauge factor of about −10.

**Figure 9:**
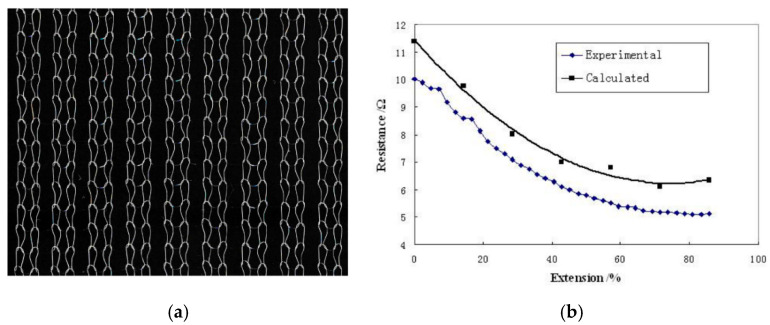
(**a**) 1 × 1 rib knitted structure; and (**b**) its elongation behaviour [55].

Yang [55] modelled the electrical performance of knitted 1 × 1 rib structures. These structures showed a resistance decrease while being stretched, an effect which was well predicted by their model (see Figure 9). The gauge factor for the first 20% stretching turned out to be close to −1.1. 

Li and coworkers [56] measured the confining pressure and electrical resistance of fabrics manufactured with eight different knitting stitches. Since their focus was on the confining pressure they did not mention the resistance changes with strain. Similarly, in a later work [57] they used silver coated polyamide yarns (Statex) of 0.295 mm diameter and 100–200 Ω/m conductivity and determined the electromechanical behaviour of (i) single yarns, (ii) looped yarns, and (iii) an intarsia knitted yarn structure (together with a 58 tex cotton yarn). In that paper, however, they only reported resistance changes versus applied load (and not strain) so gauge factors could again not be calculated.

#### 4.1.2. Strain Sensitive Knitted Structures (2010–2020)

Zieba [58] constructed a knitted respiration sensor consisting of a cotton yarn base fabric a 20 mm band of knitted silver plated polyester yarns (Xsilver, China). No details about knitting and gauge factor were reported but the respiration measurements results appeared reasonable.

**Figure 10:**
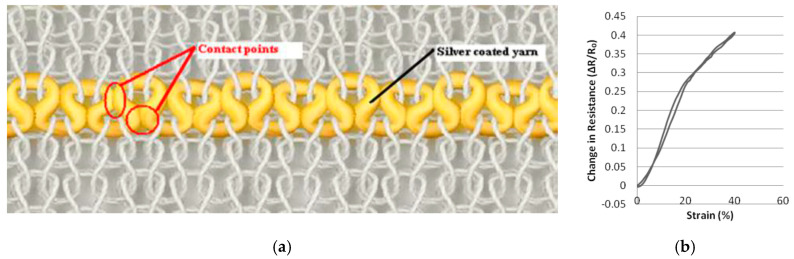
(**a**) Loop-wise embedded conductive yarn in knitted interlock structure; (**b**) electromechanical response [38].

In a series of publications the group of Ozgur Atalay (University of Manchester) studied the behaviour of knitted strain gages in more detail. In their 2013 study they used a silver coated nylon yarn (235 dtex, 200 Ω/m) plus several elastomeric yarns and varied the input tension of the elastomeric yarn to produce fabrics with different compactness [38]. The conductive yarn was embedded in the interlock base structure in a series of single jersey loops located only on the technical face of the fabric (Figure 10). The run-in tension was varied between 0.062 and 0.125 cN/tex. For each of the three fabrics they determined the wale and course density, stitch length and Tightness Factor (Equation (3)). They observed a bilinear electromechanical response with a gauge factor 3.7 below 19% strain and 2.2 above, as well as hysteresis effects. The most tightly knitted structure had the highest linearity (*GF* = 0.75). The fabrics could be extended up to 350% before breaking and were seen to be stable during cyclic testing. With the increase of fabric tightness the relaxation effects increased slightly (from 4 to 16%) [38].

In a follow-up study [59] they used a silver coated nylon yarn (Swicofil, 2 ohm/cm) and three types of Lycra base yarn covered with wrapped nylon filament (800, 570 and 156 dtex, 8 cN feeding tension). The 800 dtex Lycra yarn was studied in more detail and the conductive yarn feeding tension was varied from 5, 10, to 20 cN. It showed that at lower yarn feeding tension the loop structures were looser and the conductive contact area was increased. The 20 cN fabric showed lowest hysteresis and a bi-linear electromechanical response (*GF* = 1.86 up to 18% and 0.68 up to 40%, Figure 10b). Increasing the number of conductive courses (separated by non-conductive material) did not increase the performance due to increased tendency of the fabric to buckle. As before, the sensors were stable and showed a low drift. In their 2015 paper [60] they optimized the knitting of multiple line conductive samples and produced a sensor for respiration monitoring which was linear of to 8% with *GF* = 3.44 and which did not show hysteresis (800 dtex Lycra base yarn with 235 dtex silver plated nylon, *TF* = 1.39).

In their last study [61] they compared two types of conductive yarns (Bekinox polyester blended stainless steel and silver plated nylon), plain versus interlock base structure and elastic versus non-elastic base structure yarn. During knitting, conductive and elastic yarn are fed to same needle (plating technique) to be able to shield the back of the sensor from skin contact. From their electro-mechanical tests, it turned out that sensors with a non-elastic plain knitted base structure showed a negative gauge factor whereas elastic structures resulted in a positive gauge factor. They attribute this difference to the elastic tension which initially compressed the conductive loops in the elastic structure but which is absent in the non-elastic case. All of their results on these types of sensors, however, showed a large scatter and reproducibility, which is probably due to the lack of sufficient contraction in the sensor area compared to the base structure which may lead to buckling of the sensor part. They explained the unreliable response of their stainless steel sensors to the irregular orientation of the steel fibers within the structure. Reported gauge factors in their paper ranged from −0.7 to 1.05.

A systematic study on knitted sensor performance was conducted by the textile group of the Niederrhein university [62]. This group studied the effect of knitting structure on the strain sensing capability and considered the following parameters:

- Stitch type: double face, single face, Milano rib and full cardigan;
- Stitch cam settings NP = 9.5 (small), 10.5 (medium) and 11.5 (large);
- Conductive yarn type: four types of S-Shield PES and cotton blended stainless steel yarns (single thread, two thread; 50/2 with 20% steel fibers or 15/1 Nm with 50% steel);
- Fabric orientation: 0, 30, 45, 60, and 90°.

**Figure 11:**
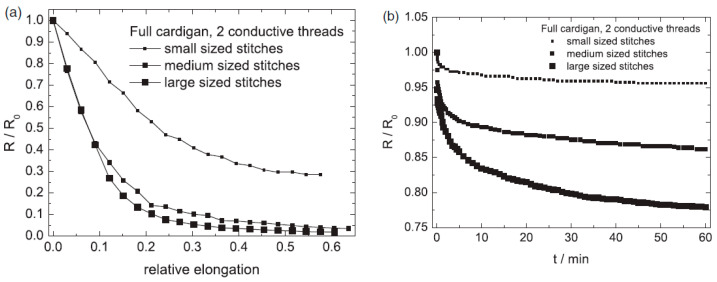
Effect of stitch size on electromechanical behaviour of full cardigan fabrics. (**a**) With respect to strain and (**b**) with respect to time [62].

They calculated wale and course densities as well as the loop lengths, which would allow them to relate the observed electromechanically behaviour to textile parameters like the tightness factor. Regarding the stitch types they observed that the single face fabrics often showed oscillatory time dependence and that the Milano rib structure had a rather small sensitivity. The full cardigan and the double face structures gave the best results (Figure 11).

Figure 11 also shows that for these knitted structures the resistance decreases when stretched (negative gauge factors) and that after the first 10% stretch the sensors rapidly became less sensitive. Furthermore, the figure shows that the smaller stitched structures had a considerable lower gage factor than the medium and larger ones (−2.2 versus −5.8, as obtained over the first 10%). On the other hand, the signal decrease over time of the smaller stitched structures was considerable better than the more dense structures (Figure 11b).

**Figure 12:**
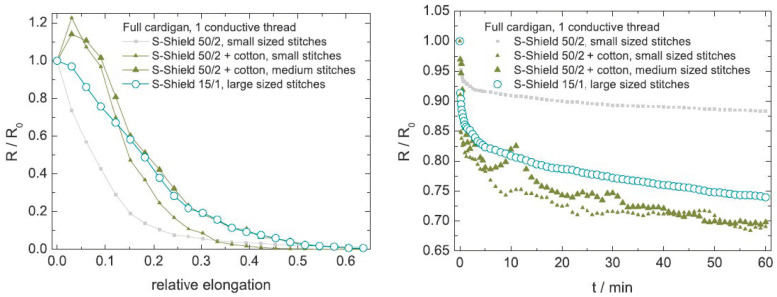
Effect of yarn materials [62].

The study of Ehrmann et al. [62], showed that the difference between the 50/2 conductive yarn containing 20% steel filaments and that of the 15/1 yarn with 50% steel was not large, as depicted in Figure 12. The 50/2 yarn had a smaller working range (up to 15 to 20%) but also a considerable smaller relaxation. More interesting to note is that the blending in of cotton threads resulted in undesirable (first increase, then decrease in resistance) as well ambiguous results (peaks and scatter in relaxation experiments). This confirmed the conclusions of Atalay et al. [61] that mixed filament conducting yarns were unsuited for constructing knitted strain sensors. The authors also showed that when elongated in wale direction (90°) the sensitivity increased at the costs of a decreased working range (limited to 20%) and relaxation behaviour. Interestingly enough, all tests at 30, 45, and 60° closely followed this wale direction behaviour.

Wang et al. [63] proposed an electrical resistive mathematical model using an empirical relation for the inter-loop resistance increase during stretching and validated their model with measurements at 2, 5, and 10% elongation. Their results showed linearity of the knitted sensors up to 5% and gauge factors of 5 to 10. Unfortunately, most experimental conditions were not reported and a good comparison between experiments and model was missing.

**Figure 13:**
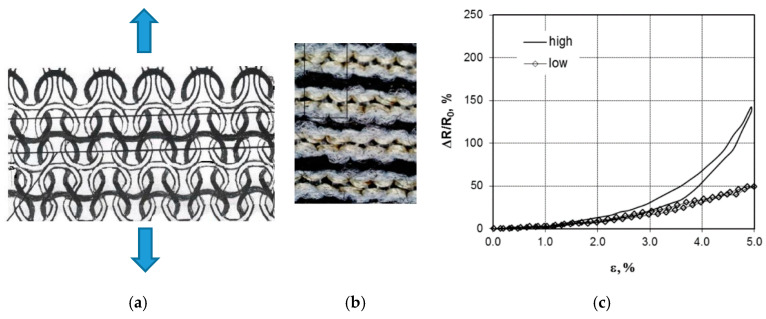
(**a**,**b**) Alternating courses of conductive and non-conductive yarns; (**c**) resistance change [64].

Oks [64] knitted plain stich sensors with alternating conductive and non-conductive courses (Figure 13). Due to the compressive effect of the elastomeric yarns the conductive courses made resistive contact when unloaded but separated gradually when loaded (in wale direction). For the experiments they used

- Base yarn: Elastane/polyester 22/78 yarn
- Function non-conductive yarn: 25 Tex cotton
- Functional conductive: Shieldex dTex110 and dTex110*2

These sensors work well for strains up to 5 or 10% and can be designed such that the resistance increases to infinity if the strain exceeds a certain value (if the conductive courses are separated too much, see Figure 13). This type of design however seems less suitable for work ranges above 20%. The sensors showed hysteresis when the knitting was done at high course density (16.8/cm). When knitted with lower course density (15.2/cm) the hysteresis was almost absent. The resistance change with applied strain curve was non-linear (Figure 13c), but when evaluated over the first 5% strain the gauge factors varied between 20 and 42. If the 110*2 double yarn was used the linearity was better but the gauge factors were a bit lower (10 to 30). The sensors were seen to be stable during 10 cycles of loading. They presented knitted sensing gloves and socks but did not discuss their properties.

**Figure 14:**
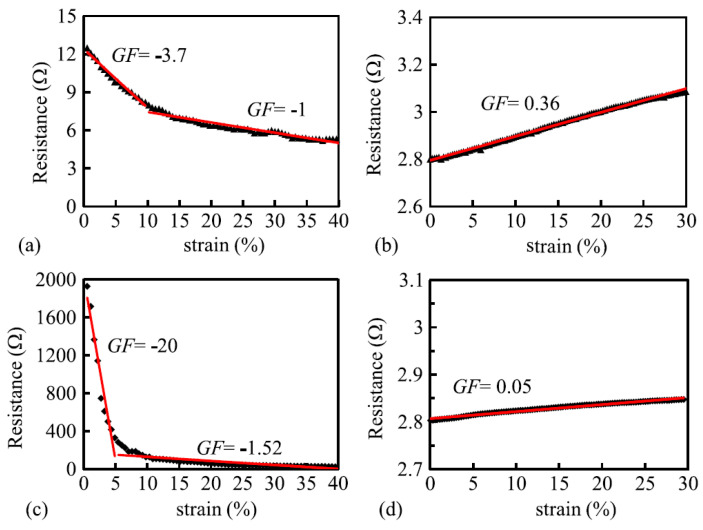
Course-wise response of (**a**) Cotton-steel knitted sensor and (**b**) nylon plated material; Wale-wise (transverse) responses of (**c**) cotton-steel and (**d**) nylon plated material [65].

Xie [65] fabricated a yarn consisting of 50% cotton and 50% 8 μm thin stainless steel fibers which looked and felt like cotton and which were robust and washable. Jersey knitted sensors showed a high, negative gage factor of −20 when loaded in wale direction but after 5% strain this diminished to −1.52 (Figure 14c). Due to this large change this type of sensor is in its current state of less practical use but may show to have a large potential when improved. In course direction the gauge factor changed from −3.7 to −1. As a comparison they also tested a sensor knitted from silver plated multifilament nylon (110 dtex/40f). These sensors were linear up to 30% strain and had gauge factors of 0.36 (course direction) and 0.05 (wale direction, Figure 14d). This once again shows that silver plated nylon yarns are much more suitable for sensor construction than stainless steel filaments blended with non-conductive material. 

**Figure 15:**
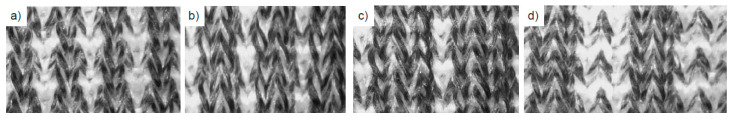
Images of knitted rib structures. (**a**) 1 × 1, (**b**) 1 × 3, (**c**) 1 × 2 and (**d**) 2 × 2 structure [66].

Rib structures have the tendency to contract laterally, resulting in a pattern in which knit stitches come forward and purl stitches recede. They provide highly elasticity and are therefore often used for cuffs and edges that require form-fitting. During stretching the vertical rib structures unfold which makes them an interesting candidate for (large deformation) stretch sensors. A detailed study on the effect of the rib structure on the electrical behaviour was performed by Raji [66] for a future application as respiration sensor in a Smart Bra. They used a silver coated nylon yarn (77 ohm/cm resistivity) as a conductor and both bare strand (BS) and nylon covered yarn (CY) spandex as the elastic yarns. The four types of mock rib structures studied are shown in Figure 15. They reported the main textile parameters (wale and course density and fabric dimensions) as well as the resistance changes during cyclic loading. Their main findings were: Gauge factors of samples with the bare strand elastane (BS) had systematically higher values (*GF* = 2.4, 2.8, 2.3 and 2.2 for the 1 × 1, 1 × 3, 1 × 2, and 2 × 2 ribs) than those of the covered yarn elastane samples (*GF* = 1.25, 1.5, 1.6, and 1.4). The sensor performance of the CY 1 × 1 and 1 × 2 as well as the BS 1 × 3 samples were seen to decrease somewhat after 50 days of testing. Unfortunately, no direct resistance versus strain plots were reported so no conclusion about linearity can be given. 

**Figure 16:**
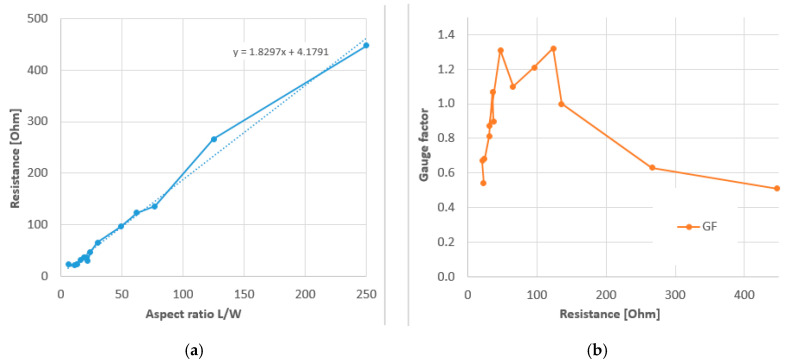
(**a**) Resistance and (**b**) gauge factor rectangular knitted sensors of different length and 6width; Based on [67].

In a subsequent paper they selected the CY 1 × 1 structures and varied the knitted sensor geometry for aesthetic reasons [67]. They concluded that complex shaped samples delivered poor repeatability and noisy results, probably because of the truncating of conductive yarns inside the non-conductive gauge areas. An extended test series with 14 rectangular samples with different aspect ratio showed first of all that the gauge resistance increased linearly with the aspect ratio, as expected from the resistance expression Equation (7). Not shown by the authors but based on their data, the surface resistivity could be determined as 1.83 Ω/sq (see Figure 16). Combining this with Equation (7) and the given linear resistivity of 77 Ω/cm this implies a fabric thickness of 0.24 mm, which could be realistic. Secondly, their results showed that the gauge factor of the rib knitted sensors have a maximum between 40 to 120 Ω. They reasoned that the decrease in gauge factor below 40 Ω was due to an increased resistive heating resulting in a less efficient sensing and that for too high resistances the current becomes too low to give reliable results. Although the latter interpretation is doubtful (other studies worked with sensors of several kΩ [50]), the idea that gauge factors show a maximum depending on their geometry is interesting to further explore for other knitting structures.

Ou et al. [31] used a 1000 Ω/m 450 denier (500 dtex) silver plated nylon blended with non-conductive yarns and an interlock knit structure. They first knitted a series of rectangular sensors with aspect ratios varying between 0.31 and 320 and confirmed the linear scaling with *L*/*W*, as expected from Equation (7) and also found in Figure 16. The surface resistivity was 2.63 Ω/sq, which is of the same order as the value for the study discussed above [67]. The sensitivity to stretching was only shown for a single sensor geometry (with 60 Ω initial resistance). The sensor response was more or less linear but the strain range and sensitivity could not be determined since the sample length was not reported.

#### 4.1.3. Modelling of Knitted Sensor Electromechanical Behaviour

About the physical interpretation for the observed decreasing and increasing resistance-strain curves there seems to be a general consensus in the literature, although they have never been listed together. Summarizing the literature, we can discern the following four distinct stage during the stretching of a knitted conductor:

- Stage 1: Reduction of contact points: the opening of the structure (gradually) disconnects contacting loops which were connected in the initial state due to elastic contraction of the base structure. This is typical for the first 10–20% stretching and causes a resistance increase;
- Stage 2: Yarn slippage and shifting of contact points due to a rearrangement of the knitted structure. This results in a larger yarn length (and resistance) between a fixed number of contact points and can be a dominating effect for high resistance yarns. Between 10 and 40% strain;
- Stage 3: Increased contact pressure between hooked conductor loops. This effect starts to play a role for strains above, say, 20% and causes the resistance to decrease. In bare metal conductive yarns this may start earlier and is the dominating effect;
- Stage 4: The stretching of the conductor yarn segments itself. This occurs when all slack is out of the system and loop segments have straightened. Depending on the structure this may start above 50 to 80%. For metal coated and carbon filled yarns this results in an increase in resistance whereas for yarns consisting of metal filaments blended with non-conductors the resistance decreases due to the squeezing effect.

**Figure 17:**
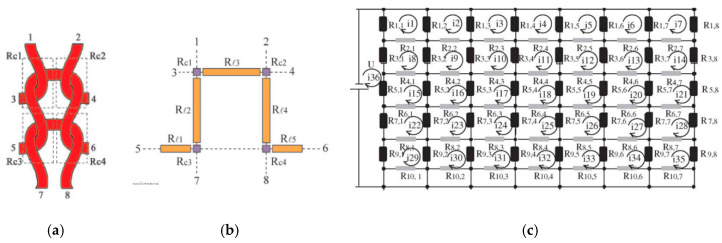
(**a**,**b**) Unit cell of knit [68]; (**c**) Resistor network approach [53].

The modelling is done by first calculating the resistance and then separately addressing the above four stages. We will not discuss the literature on resistance modelling in detail but in general this is based on first considering the resistances of loop segments and contacts in a unit cell (Figure 17a), followed by calculating the equivalent resistance of a network of such unit cells (Figure 17b). This always led to unnecessary large and complex expressions which are difficult to handle and did not give much insight. Refreshing is therefore to follow the way of reasoning of Li et al. [68]. These authors take into account that most segment resistances are of equal value and that adjacent unit cells therefore act as balanced Wheatstone bridges through which no current flows. 

**Figure 18:**
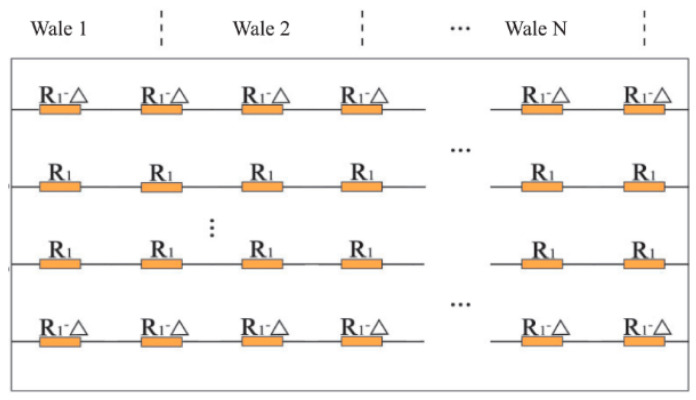
Simplified equivalent network model [68].

The current then in fact appears to flow only along the courses without any interaction between adjacent courses, which considerably simplifies the network model (see Figure 18). Note that the upper and lower courses of the conductive area have a resistance which differs from those in between due to the fact that the outer part of the loops there are not in in contact with other conductors and miss the two contact resistance points (points *R_c_*_1_ and *R_c_*_2_ Figure 17b). 

For structures with more than three courses the equivalent resistance then becomes [68]

(8) $$R_{eq}=\frac{2n_{w}R_{1}}{n_{c}+K};\;K=\frac{R_{1}+R_{1∥3}}{R_{1}-3R_{1∥3}}$$

in which both *R*_1_ and *K* depend on the contact resistance. It is clear that for large enough number of courses the resistance scales with the ratio of the number of wales *n_w_* and courses *n_c_*, or as the length over width ratio *L*/*W*, as seen in Equation (7).

Next the question is how to calculate the resistance increase due to stretching. Although some studies on the topic are reported, a generally accepted theory is not available yet. Stage 1 (the gradual decrease of contact points) is discussed by e.g., [60] who take into account Holm’s contact pressure theory. Xie et al. [69] address stage 2 and assume that during stretching the yarns slide, resulting in longer loop segments in tensile direction (*R*_*l*3_ in Figure 17b). The loop segment length changes during stretching were empirically determined and with this and their (complicated) network model they could correctly predict the overall resistance change due to stretching. In other works often the empirically determined contact resistance variation with pressure is used to describe stage 3 [53]. The fourth stage can be taken into account by first estimating how the yarn stretching relates to the macroscopic strain in the structure and then using the (measured) resistance increase of the conductive yarns.

### 4.2. Stitched Sensors

With knitting the sensor placement and orientation is limited in flexibility because of the fact that many hidden conductive tuck yarns are needed to realize more complex shapes as well as orientations different from wale and course directions. Although these hidden yarns are not visible, they tend to corrupt proper functioning of the sensor [67]. Stitching, on the other hand, can be directly applied to a finished garment and is not limited on orientation direction. Moreover, stitched sensors have no perceptible compromise for user comfort and a minimal impact on garment production [70]. The sensor response however relies heavily on the physical characteristics of the textile substrate to which it is stitched.

Much of the work on stitched strain sensors has been done by the group of Dunne (University of Minnesota). In their first work on the topic Gioberto and Dunne [70] present a top-thread cover stitch stretch sensor using a silver plated nylon yarn (Lamé Lifesaver, 0.81 ohm/cm) on a 98% polyester/2% Lycra jersey knit fabric. The sensor had a work range of about 12% and a gauge factor of about 0.54. In their 2013 paper [71], they tested overlock-stitched strain sensor using a Shieldex 235/34 silver coated nylon yarn (50 Ω/m) and five types of jersey knit fabrics with different elastomeric content. Their sensors turned out to have a work range between 18 and 29% and relatively small drift and hysteresis. Gauge factors were not reported but can be estimated to vary between 0.48 and 2.2. The sensor with the highest work range was the one stitched on fabric consisting of 82% nylon and 18% spandex. In another paper [72] they compared overlock and bottom thread cover stitches using two, four, and five-ply silver plated nylon yarns (Shieldex 177/17, Shieldex 235/34 and a custom made thread). Their results showed that sensors fabricated with the two-ply thread were less repeatable and showed significantly more noise that the other sensors. The working range of the top-thread cover stich and overlock sensors are limited to 5–10% and 20–25%, respectively. The bottom thread cover stitch sensor, on the other hand, showed decreasing resistance upon stretching and had a working range to above 40% strain. They tested their sensors on a leotard and two types of shirts for a spinal posture sensing application. The sensors were stitched to the dorsal side along the spine and insulated with 3M7012 stitchless bonding film. Those tests showed that the T-shirts were less suitable for posture sensing since during multiple bending they tended to ride up on de body and do not slide back.

**Figure 19:**
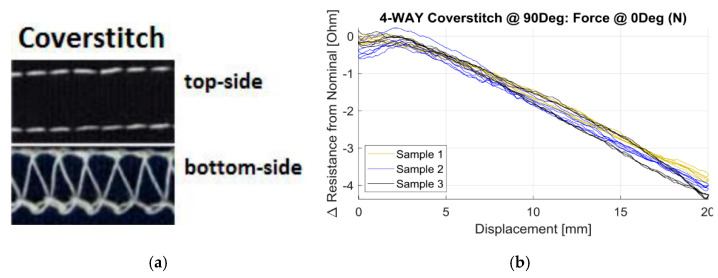
(**a**) Coverstitch structure and (**b**) resistance versus displacement curve [37].

The response of stitched sensors depends on the stitch structure and the anisotropy of the base or substrate material whereas for the suppression of hysteresis it is important how the (elastic) response of the substrate or surrounding yarns are. In [37] the effect of the substrate materials on strain gauges embroidered at different stitch angles was investigated. The authors used a two-way and four-way stretch weft knit as well as a double knit structure and Shieldex 235/34 four-ply silver plated nylon as the conductive yarn. The stitches considered were the ISO401 chain stitch and the ISO406 bottom cover stitch. They showed that the chain stitch had a considerable effect on local fabric stiffness (stiffness increase of 12% at 0° orientation and 200% at 60°). The sensors were linear up to at least 30%. The highest gauge factor was obtained for the two-way knitted fabric with chain stitch (*GF* = −2.24) and the lowest was for the two-way knit fabric with coverstitch (−1.01). Remarkably the gauge factors did not depend much on the sensor orientation for all combinations tested. If on the other hand the force direction was varied the effects were larger. The transverse sensitivities of the two-way knit fabric with coverstitch sensor were unacceptable high (65% up to 98%), the two-way chain stitch and the four-way chain stitch combinations showed much lower values (4 to 12%). The chain stitch is thus the preferred stitch geometry. A typical example is shown in Figure 19.

Ruppert-Stroescu and coworkers [73] investigated the sensor properties of three conductive yarn types Shieldex 117/7 (a silver coated two-ply nylon, 985 Ω/m), Shieldex 234/34 (a four-ply silver coated yarn, 80 Ω/m) and Lame Lifesaver yarn (a three-ply silver coated yarn with 65 Ω/m resistance) on plain woven cotton fabric (muslin). Furthermore, they considered five stitch types (310, 304, 315, and 401, and adapted 304) with fixed stitch densities. It turned out that for some of the stitch/wire combination the resistance after stitching was considerably less than that of the bare wire (all per unit width) whereas for most other combinations this was not the case. Elongation tests were only performed with the bare threads so no gauge factors of the stitch types could be determined.

Vogl [44] stitched conductive silver plated yarns on a small stretchable band and thus created stretch band sensors which can be used as future user interfaces. To construct this, they used silver plated nylon (Inventables, triple twine, 30 Ω/cm), a highly stretchable 70% polyester/30% elastodiene fabric and a zig-zag stitch with 3.3 mm width. The response during stretching however appeared to be quite non-linear. 

Another stitched sensor study was performed by Greenspan [74] with the aim to apply it for unobtrusive human movement monitoring. They selected a silver plated 22/1 dtex nylon yarn, since preliminary experiments showed that it had the highest strain sensitivity. They further considered three fabric substrates (nylon/10% spandex, polyester/10% spandex and cotton/5% spandex). The stitch geometries studied were 304, 402, 406, and 514. The 514 stitch (overedge stitch) showed negligible response to stretching (*GF* close to 0) and was not further analysed. Note that such stiches however can be useful as interconnects in which resistance changes are often undesired. The best sensors candidates were the Zig-zag (304) and the chainstitch (402) on a cotton/spandex substrate. The Zig-zag on polyester/spandex combination however showed better repeatability after cyclic testing. The gauge factor as derived from their graphs was near 1.0.

**Figure 20:**
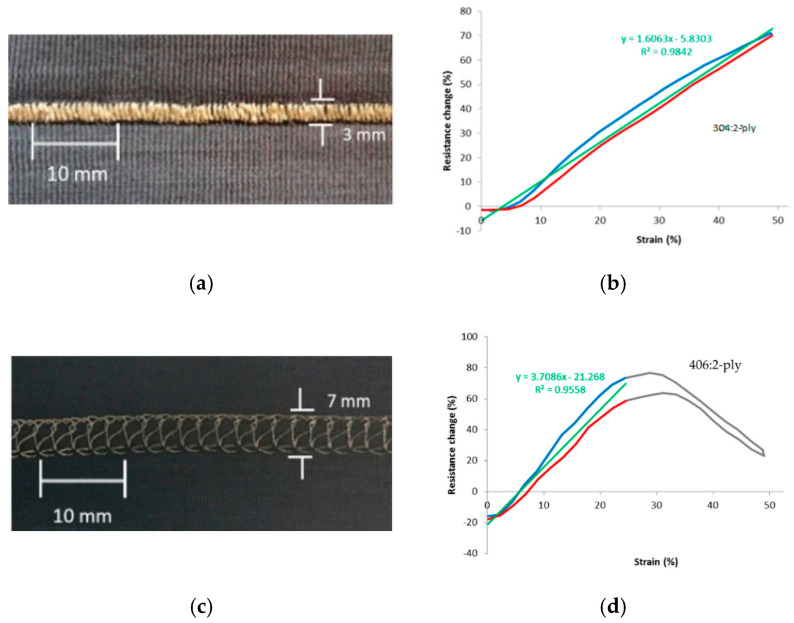
(**a**) Zigzag stitch and (**b**) corresponding resistance plot; (**c**) Coverstitch and (**d**) corresponding resistance plot [42].

As mentioned above, the response depends on the substrate fabric, the yarn, as well as on the stitch structure. If the substrate shows hysteresis after stretching, the fabricated sensor will also. Tangsirinaruenart [42] therefore suggested to first select the substrate fabric and then consider the yarn and stitch geometry. They evaluated six fabrics, two conductive threads (four-ply Shieldex 234/34 and two-ply 117/17) and four stitch types (numbers 304, 406, 506, and 605). Preliminary experiments showed that the fabric with the highest elastic recovery (93%) was a single jersey nylon (two-ply, 4.44 tex) with 25% spandex (7.78 tex) combination. Using that fabric they showed that the 304 stitch (the Zig-zag lock stitch) in combination with the two-ply wire gave the best results: gauge factor 1.61, working range 50%, good linearity, low hysteresis, and good repeatability (Figure 20a,b). A more typical response cure is shown in Figure 20d showing a resistance maximum and limited working range. Note that their experience with the two-ply conductive yarn is different from that reported by Gioberto and Dunne [72] who concluded that these yarns resulted in noisy and less repeatable sensors.

## 5. Discussion

In the above sections we have discussed all studies that involve the development and testing of knitted and stitched textile sensors. In the following two sections we summarize the performance data obtained from those studies with the aim to be able to facilitate the selection of the most suitable sensor design for a specific project. As performance indicators we selected the initial resistance, the gauge factor, working range and the strain related hysteresis (as defined by Equation (6)). If the chosen indicators were not calculated by the authors themselves, they were extracted from the resistance versus strain graphs in the papers. In this way we hope to provide a clearer and more uniform comparison of the sensor performance.

While selecting a sensor design the working range is the first indicator to pay attention to. Sensors needed for respiratory sensing need to detect chest expansions with strain of only 5%. Taking into account that the garment needs an additional 5% pre-strain when fitting around the chest, a working range of 10% seems sufficient for these purposes. The gauge factor however should be high enough such that the 5% strain variation is still measurable. Since for this application only the correct detections of peak-to-peak distances are relevant, the linearity of the response curve, hysteresis, and signal drift are less important.

For movement and activity classification purposes the limb and upper torso deformations need to be detectable with sufficient accuracy, resulting in a desired working range of 30 to 35% and a preferably low hysteresis value. Applications like the monitoring of sports kinematics, rehabilitation, and movement tracking for gaming or VR require much higher demands on hysteresis and drift stability. Demands which probably cannot be met with the sensors presented in this review. Similarly, long term monitoring of small strain changes, as, e.g., needed for edema sensing, are currently still challenging since they require low drift and hysteresis levels.

Applications in the human–computer interaction field, on the other hand, are much less demanding since in that case the sensing thát a surface is being stretched is already sufficient.

### 5.1. Knitted Sensors

In Section 4.1 we discussed 13 studies in the first 10 years of this century and another 17 papers in the decade that followed. As could have been expected, the early papers were more about demonstrating that knitted conductive yarn structures are able to sense strains whereas in later years the focus was more on the improvement of the sensor performance and therefore contained much more details about aspects like initial resistance, gauge factor, working range and hysteresis. Table 1 summarizes these performance indicators. In the table we ranked the studies as much as possible in chronological order but kept publications from the same author groups together. Based on Table 1 we can make the following observations.

#### 5.1.1. Initial Resistance

The initial resistances of knitted sensors are seen to decrease from kOhms in the early works to around 10–50 ohms in later years (Table 1). This decrease in resistance is probably directly related to the introduction of yarns with better conductivity in the early 2010s. The question we want to pose here is how beneficial this trend of lower resistances is for the performance of the sensors. It can be argued that the gauge performance is likely to decrease if the gauge resistance drops below a level of about 10 ohm because of the increased influence of contact resistances. If we assume that the lead wires which connect the strain gauge to the data processing and power units have a resistance of a few ohms (depending on the length and conductivity), the resistance of the gauge should be much larger (e.g., a factor 10) to avoid that resistance changes in these lead wires and contact points during body movements contribute too much to the signal. This agrees with the observation of [67] that sensors with lower resistances showed a noisier behaviour. Moreover, low resistance gauges consume more power. Measurements are usually done by supplying a constant voltage and measuring the changes in current. For a sensor with 10 ohm resistance and 5 Volt applied, the power consumption amounts to *P* = *V*^2^/*R* = 2.5 W (and *I* = *V*/*R* = 500 mA), which is quite high. A sensor with a resistance of, e.g., 1000 Ω, is much less sensitive to lead wire effects and consumes 100 times less energy. From this we conclude that in order to suppress noise from fluctuating contact resistances and in view of energy efficiency future strain gauges should aim at initial resistances in the range of 100–1000 Ohm rather than the current practice of 10–50 Ohm.

#### 5.1.2. Conductive Yarns

The best results seem to be obtained with silver plated nylon yarns (i.e., Shieldex or Lamé Lifesaver yarns). Most often used is the Shieldex 234/34 four-ply yarn from Statex. Bare metal fiber yarns and metal fiber yarns blended with non-conductive cotton or polyester (Bekinox) have a negative gauge factor. The blended yarn combinations however are much less suitable for application as sensor since the irregular arrangement of the non-conductive thread and stainless steel filaments result in noise and less reproducible results. Greenspan [74] argues that the yarns should be of 140–700 dtex and have linear conductivities above 1 Ω/m.

#### 5.1.3. Performance Indicators

The gauge factors reported in literature range from 0.3 to 17 and can be both positive and negative. The value of the gauge factor itself is in fact not that important as long as it allows to collect data with low enough noise levels, which is typically true for gauge factors above one. Note that the development of sensors with extremely high gauge factors is useless for on-body applications as we are discussing here, since we do not need to be able to detect ultra-low strain levels, as opposed to typical mechanical engineering applications. In Table 1 we highlighted those strain sensors which had a working range of at least 30% and had hysteresis values below 0.10. The three best performing sensors are all variations to the method developed by Atalay et al. [38] which utilizes a single course, loop-wise embedded conductor yarn, as discussed in Section 4.1.

The reported working ranges vary from 8 up to 80%. Typical values are around 30 to 40% which is just enough to detect body movements like elbow and knee deflection. Hysteresis values were more difficult to obtain but all values were in the range from 0.07 to 0.14.

### 5.2. Stitched Sensors

The work on stitched textile strain sensors started much more recently with the work of Gioberto and Dunne [70]. In all studies silver-plated nylon yarns are used as conductors with the Shieldex 117/17 and 234/34 types as the preferred candidates. For stitched sensors the substrate material is an additional parameter with effect on the sensor performance. It is important to realize that for optimum sensor performance care should be taken in selecting a suitable substrate fabric with low mechanical hysteresis. The best performing sensors have jersey knitted substrates containing 10–25% elastomeric (Spandex) yarn. In case the sensors have to be integrated in a different, less elastic, fabric it may be useful to integrate patches of a more elastic substrate with stitched-on sensor in the garment. An important conclusion from the work of Dupler [37] is that the sensitivity did not vary much with the sensor orientation and is always largest in sensor direction. This in fact simplifies the positioning of sensor elements on the textile. The 406 bottom cover stitch and the 304 Zig-zag stitch seem to give the best results, as shown in Table 2. The gauge factors are typically in the range of 1 to 1.6 and can be either positive or negative, depending on whether the resistance changes due to the gradual breaking of loop–loop contacts during stretching (positive gauge factor) or due to the increased squeezing contact between adjacent conductive lines (negative gauge factor). For the functioning of the sensor both variants can be expected to work well. Compared to the previous evaluation of knitted sensors we can see that the working range for stitched sensors is usually higher (often exceeding the 50%), whereas hysteresis levels are similar. The best performing sensor can be considered as the bottom coverstich sensor of Dupler [37] which has an extremely low hysteresis value of 0.02 and thus outperform the knitted sensors in this respect.

Note that there is an inconsistency in the naming of the stitch geometries in literature. The 406 type is according to the ASTM-D6193 classification the two-needle bottom cover stitch but is referred to by [42] as the two-needle rear side chain stitch. In their recommendations [42] suggest to increase the stitch density in order to improve the performance of the strain sensors. This recommendation is based on the fact that the initial gauge factor of stitched sensors is mainly determined by the gradual opening of loop-to-loop contacts [70], a process which can be expected to proceed more uniformly for larger stitch densities.

## 6. Conclusions

### 6.1. Sensor Selection (“Do’s and Don’ts”)

From the above study we can conclude that

- Knitted strain gauges are best produced with silver plated nylon yarn as the conductor thread. Blended yarn combinations like stainless steel/polyester should be avoided since this results in less reproducible sensor performance
- The loop-wise embedding configuration of the conductive yarn as used by Atalay [38] results in the best overall sensor performance
- Knitted gauges of non-rectangular shapes suffer from sensor noise and poor reproducibility due to the need to truncate conductive yarns outside the sensor area [67]
- In order to reduce hysteresis and relaxation effects in stitched strain sensors it is important to use a highly elastic substrate fabric with minimum mechanical hysteresis.
- The bottom coverstich sensor of Dupler [37] has hardly any hysteresis, and is thus the preferred configuration. As alternative the zig-zag stitch also performs well
- The sensitivity of stitched sensors can probably be improved by increasing the stitch density

Researchers or manufacturers aiming to fabricate garments with embedded strain sensors using currently available materials and technology, have the choice between knitting and stitching of conductive yarns in their fabrics. Both of them do not compromise comfortability and washability and can result in robust and easy to manufacture sensors. Stitched sensors are simple to apply and are most likely the preferable choice for prototyping sensor garments. As mentioned above, when constructing stitched sensors, care should be taken to select a substrate with good elastic properties. The bottom coverstitch and the zig-zag stitch are the best candidates for sensors with low hysteresis values. Note that for zig-zag stitches to perform well it is required to have a high enough stitch density such that consecutive loops can contact in the unloaded state.

Knitted sensors can be completely embedded in the fabric structure and can be fabricated in a single process step. They are therefore the preferred option when the sensor garments have to be mass produced. The best options here are to use silver plated nylon yarns, in combination with the loop-wise embedding configuration proposed in [38].

### 6.2. Topics for Future Research

In most of the studies discussed above textile sensors were created by constructing rectangular strips of which the resistance changes uniformly upon stretching. There were however two alternative ideas proposed with different sensing mechanisms. Oks and co-workers [64] for example made structures consisting of separated conductive lines (courses) which touch when unloaded but separate when loaded. This resulted in an a-typical sensor curve in which the resistance rapidly rises above a threshold value which depends on the knitting structure. Such constructions can serve as a starting point for designing knitted structures in which the resistance increase, e.g., occurs in a stepwise way by introducing conductive bridges of different size in the structure. This type of parallel line structures can also be helpful for developing direction sensitive sensors.

Another interesting idea for strain sensing in textiles is the inductance idea of Wijesiriwardana [46]. They propose to knit a structure of parallel lines or coils and use the change in inductance as a measure of deformation. This technology could be interesting since it has the option of adjusting the sensing frequency to suppress noise (see also the study of Dhawan [36]).

The time-dependent sensor responses like the dependency on the loading velocity, the signal relaxation, drift, and hysteresis largely complicate the data analysis process. One should therefore first try to reduce these effects as much as possible by selecting an appropriate substrate and sensor structure. If one then still wants to correct for these effects it may be useful to use the modelling and characterization techniques developed to describe polymer viscoelasticity since this provides a consistent framework for describing time dependent effects with a limited number of parameters. In that way it should be possible to have a simple unified model which both predicts relaxation, hysteresis and drift with a single set of parameters. More information can be found in standard viscoelasticity textbooks.

An idea for further study is to repeat the aspect ratio of knitted sensors for different stitch types and observe if also a maximum in the gauge factor is obtained as reported by Raji [67]. In addition, our hypothesis that gauges with low initial resistances are more susceptible for contact point related noise can be verified with a study in which the yarn conductivity is systematically varied. Finally, the determination of gauge factors at temperatures different from room temperature can be used to find out if the gauge factor is affected by heating or not. Such studies are expected to contribute to a more fundamental understanding of the working principles of knitted sensors than we presently have.

Future research should focus on reliability and robustness of the sensors. In current studies the reliability is often studied by cyclic loading in laboratory test setups. This of course gives a good initial indication of the sensor stability but not about the sensor behavior during actual wearing conditions. In such situations sweating, mechanical friction and washing may lead to unexpected sensor degradation. In addition, extra research is needed regarding the interconnection of the sensors with data processing and power units since these should be unobtrusive, robust and washable as well. In particular, it can be expected that the connection points between flexible interconnect wiring and rigid power or data processing units easily fail during extensive bending such as can be expected to occur during machine washing.

The sensor types we have discussed here are good candidates to be used in future smart garments in which the sensors are combined with local energy harvesting elements and are part of a body area network, which processes the data and transmits it wirelessly to the cloud.

## Figures and Tables

**Table 1:** Performance comparison of knitted sensors. Sensors with working range above 30% and low hysteresis are highlighted.

Reference	Knit Type	Conductive Yarn ^1^	Non-Conductive Yarn	*R*_0_ (Ω)	GF (Range) ^2^	Working Range (%) ^3^	Hysteresis *H_ε_* ^4^	Remarks	Application
Farringdon 1999 [43]	-	-	-	50.000	17	40%		Sensor strips	Activity sensing
Bickerton 2003 [45]	plain	Carbon fibre	Lycra	90.000	14	40%			Body kinematics
Wije 2003 [35]	-	Carbon rubber	elastic	400.000	2.4	10%			Body kinematics
Atalay 2013 [38]	interlock	235 dtex Sil-Ny, 200 Ω/m	800 dtex elastic	166	**3.75**	**10–40%**	**0.10**	1-course loopwise embedded	Body kinematics
Atalay 2014 [59]	interlock	Sil-Ny, 200 Ω/m	800 dtex elastic	-	**1.86**	**6 to >40%**	**0.07**	1-course loopwise embedded	
Atalay 2017 [61]	plain	Sil-Ny, 200 Ω/m	800 dtex elastic	10.4	1.05	80%		noisy	
	plain	Sil-Ny, 200 Ω/m	167 dtex polyester	5.3	−0.38	20%		noisy	
	interlock	Sil-Ny, 200 Ω/m	800 dtex elastic	2.3	0.97	40%		noisy	
	interlock	Sil-Ny, 200 Ω/m	167 dtex polyester	4.6	−0.70	55%		noisy	
Atalay 2015 [60]	interlock	Sil-Ny, 200 Ω/m	800 dtex elastic	261	**3.44 (8%)**	**>40%**	**0.08**	1-course loopwise embedded	Respiration monitoring
Zhang 2005 [54]	plain	Bare SS	-	12	−8.6 (10%)	22%			
Zhang 2006 [53]	Single warp	carbon	-	15.000	−13 (2%)	8%	0.08		
Yang 2009 [55]	1 × 1 rib	Bare SS	-	11.5	−1.1 (40%)	50%			
Ehrmann 2014 [62]	full cardigan	PES/SS, 525 k Ω/m	cotton	-	−6.2 (10%)	20%		Resistance drops to zero	
	Double face	PES/SS, 525 k Ω/m		-	−2.2 (20%)	55%		Large relaxation	Respiration monitoring
Pacelli 2013 [50]	intarsia	Cf-Ny (Belltron)	Lycra	44.300	0.75 (16%)	>16%	0.14		Rehabilitation, Body kinematics
Oks 2014 [64]	plain	Sil-Ny	Elastane, PES, cotton	12	>10 (5%)	5–10%	0.10	Alternating conductive rows	Sensing glove, sock
Tognetti 2014 [75]	intarsia	Cf-Ny (Belltron)	Lycra	90.000	6.7	>10%	0.14	Single layer strip	Rehabilitation, Body kinematics
Ou 2019 [31]	interlock	Sil-Ny	Spandex, PES	60	2.3			L_0_ estimated	Human Computer Interaction
Xie 2016 [65]	plain	Cotton/SS, 500 Ω/m	cotton	12	−3.7 (10%)	40%		Custom blended yarn	Body kinematics
	plain	Sil-Ny, 500 Ω/m		2.8	0.36 (>30%)	>30%			
Raji 2018 [66]	1 × 1 rib	Sil-Ny, 7700 Ω/m	Nylon covered spandex	32.8	1.25	>10%			Respiration, smart bra
	1 × 2 rib			25.3	1.5				
	1 × 3 rib			25.9	1.6				
	2 × 2 rib			31.7	1.4				
	1 × 1 rib		Bare spandex	14.9	2.45				
	1 × 2 rib			12.3	2.85				
	1 × 3 rib			11.1	2.3				
	2 × 2 rib			15.1	2.2				
Raji 2019	[76] 1 × 1 rib	Sil-Ny, 7700 Ω/m	Nylon covered spandex	47.1	1.31	>10%		12 other sizes tested	Respiration, smart bra

^1^ Sil=silver coated; Ny = nylon; SS = stainless steel; PES = polyester; Cf = carbon filled; Cc = carbon coated; ^2^ range for which the GF approximately applies; ^3^ notation ‘>x%’ means tested until x% but possibly larger; ^4^ hysteresis in length relative to maximum length (mm/mm) or as Δε/εmax (%/%), see Equation (6).

**Table 2:** Performance comparison of stitched sensors. Sensors with working range above 30% and low hysteresis are highlighted.

Author	Stitch Type	Conductive Yarn ^1^	Substrate Fabric	R_0_ (Ω)	GF (Range) ^2^	Working Range (%) ^3^	Hysteresis *H_ε_* ^4^	Remarks	Application
Gioberto 2012 [70]	602, top coverstitch	Sil-Ny, Lamé, 81 Ω/m	-	120	0.54 (12%)	10%	-	Non-linear, low hysteresis	
Gioberto 2013 [71]	514, Overlock	Sil-Ny, 50 Ω/m	100% PES jersey	43	0.94 (10%)	19	-	*H_R_* = 0.19	
			60 cotton/40 PES	55	0.48 (10%)	21	-	*H_R_* = 0.35	
			90 PES/10 spandex	58	2.22 (10%)	17	-	*H_R_* = 3.04	
			94 cotton/6 spandex	43	1.41 (10%)	22	0.17	*H_R_* = 1.15	
			82 Ny/18 spandex	43	1.43 (10%)	29	-	*H_R_* = 0.82	
Gioberto 2016 [72]	602, bottom coverstitch	2-ply Sil-Ny Shieldex	Jersey knit elastomeric blend	2370	**−1.22 (50%)**	**>50%**	-	Noisy signal	
		4-ply Sil-Ny Shieldex	Jersey knit elastomeric blend	61	**−0.89 (50%)**	**>50%**	-	Good signal	Spinal posture sensing
Dupler 2019 [37]	406, Bottom coverstitch	Sil-Ny Shieldex	4-way PES/Spandex	10.9	**−1.15 (30%)**	**>30%**	**0.02**	*H_R_* = 0.23	
	401, Chain stitch			7.7	−1.76 (30%)	>30%	-	*H_R_* = 0.34	
	406, Bottom coverstitch		2-way PES	10.9	−1.13 (30%)	>30%	-	*H_R_* = 0.08	
	401, Chain stitch			7.7	−2.20 (30%)	>30%	-	*H_R_* = 0.34	
Greenspan 2018 [74]	304, Zigzag	Sil-Ny/PET, 30 kΩ/m	90% PES/10% elastane	7000	**1.0 (5–28%)**	**>30%**	**0.10**		Body kinematics
Tangsiri 2019 [42]	304, Zigzag	2-ply Sil-Ny 117/17	75% Ny/25% Spandex	125	**1.61 (50%)**	**50%**	**0.07**	*H_R_* = 0.09	
	406, chain stitch	2-ply Sil-Ny 117/17		71.5	3.71 (25%)	25%	0.20	*H_R_* = 2.51	
		4-ply Sil-Ny 234/34		55.6	2.71 (8%)	8%	0.55	*H_R_* = 0.71	
	506, overlock	2-ply Sil-Ny 117/17		649	0.099 (16%)	16%	0.36	*H_R_* = 0.05	
		4-ply Sil-Ny 234/34		46.7	5.16 (12%)	12%	0.51	*H_R_* = 0.54	
	605, coverstitch	2-ply Sil-Ny 117/17		240	0.21 (18%)	18%	0.04	*H_R_* = 0.59	
		4-ply Sil-Ny 234/34		39.3	1.65 (18%)	18%	0.14	*H_R_* = 0.09	

^1^ Sil = silver coated; Ny = nylon; SS = stainless steel; PES = polyester; Cf = carbon filled; Cc = carbon coated; ^2^ range for which the GF approximately applies; ^3^ notation ‘>x%’ means tested until x% but possibly larger; ^4^ hysteresis in length relative to maximum length (mm/mm) or as Δε/εmax (%/%), see Equation (6).
